# Supplementary material for: Comprehensive Annotation and Functional Exploration of MicroRNAs in Lettuce
Source: Front Plant Sci. 2021 Dec 24;12:781836. doi: 10.3389/fpls.2021.781836 (PMC8739914; doi:10.3389/fpls.2021.781836)
Supplement: Supplementary file 2 [file Data_Sheet_8.ZIP › GSM2230748.plot/Lsa-miR398a_Lsat_1_v5_gn_8_117301.1_62_TPlot.pdf]

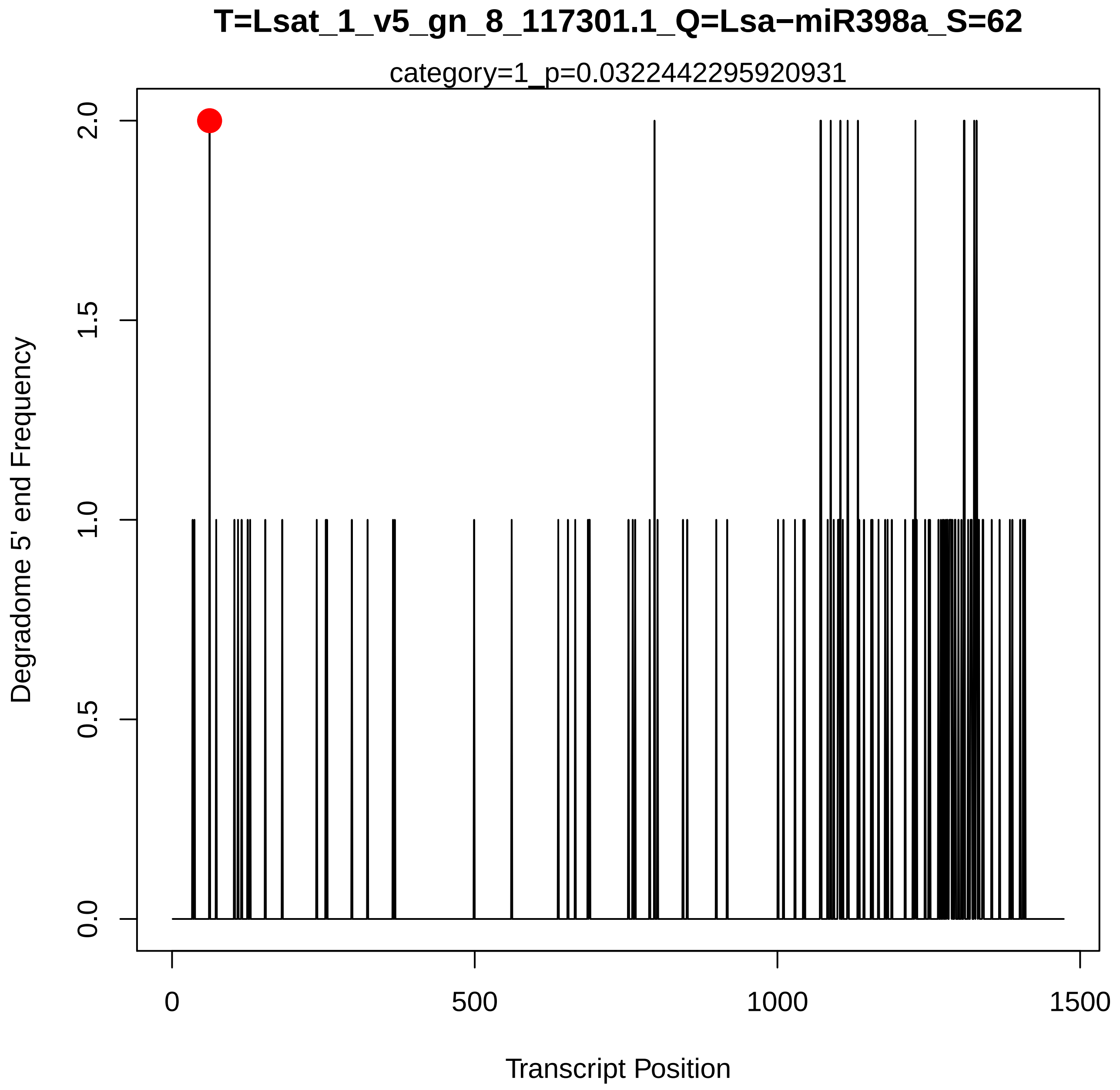
T=Lsat_1_v5_gn_8_117301.1_Q=Lsa−miR398a_S=62
category=1_p=0.0322442295920931
Degradome 5' end Frequency
2.0
1.5
1.0
0.5
0.0
0
500
1000
1500
Transcript Position
